# Electrospun Polycaprolactone Fibrous Membranes Containing Ag, TiO_2_ and Na_2_Ti_6_O_13_ Particles for Potential Use in Bone Regeneration

**DOI:** 10.3390/membranes9010012

**Published:** 2019-01-10

**Authors:** Erick Ramírez-Cedillo, Wendy Ortega-Lara, María R. Rocha-Pizaña, Janet A. Gutierrez-Uribe, Alex Elías-Zúñiga, Ciro A. Rodríguez

**Affiliations:** 1 Tecnologico de Monterrey, Escuela de Ingeniería y Ciencias, Av. Eugenio Garza Sada #2501 Sur, Monterrey, NL 64849, Mexico; A00806274@itesm.mx (E.R.-C.); mrochap@tec.mx (M.R.R.-P.); jagu@tec.mx (J.A.G.-U.); aelias@tec.mx (A.E.-Z.); ciro.rodriguez@tec.mx (C.A.R.); 2 Laboratorio Nacional de Manufatura Aditiva y Digital (MADIT), Autopista al Aeropuerto, Km., 9.5, Calle Alianza Norte #100, Parque PIIT, Apodaca, NL 66629, Mexico; 3 Tecnologico de Monterrey, Escuela de Ingeniería y Ciencias, Vía Atlixcáyotl 2301, Reserva Territorial Atlixcáyotl, Puebla, PUE 72453, Mexico

**Keywords:** electrospinning, antibacterial, cell proliferation, bioactivity, polycaprolactone, titanium dioxide, silver, sodium hexatitanate

## Abstract

Biocompatible and biodegradable membrane treatments for regeneration of bone are nowadays a promising solution in the medical field. Bioresorbable polymers are extensively used in membrane elaboration, where polycaprolactone (PCL) is used as base polymer. The goal of this work was to improve electrospun membranes’ biocompatibility and antibacterial properties by adding micro- and nanoparticles such as Ag, TiO_2_ and Na_2_Ti_6_O_13_. Micro/nanofiber morphologies of the obtained membranes were characterized by X-ray diffraction, Fourier-transform infrared spectroscopy, differential scanning calorimetry, scanning electron microscopy, energy-dispersive X-ray spectroscopy and a tensile test. Also, for this study optical microscopy was used to observe DAPI-stained cells. Membranes of the different systems were electrospun to an average diameter of 1.02–1.76 μm. To evaluate the biological properties, cell viability was studied by growing NIH/3T3 cells on the microfibers. PCL/TiO_2_ strength was enhanced from 0.6 MPa to 6.3 MPa in comparison with PCL without particles. Antibacterial activity was observed in PCL/TiO_2_ and PCL/Na_2_Ti_6_O_13_ electrospun membranes using *Staphylococcus aureus* bacteria. Bioactivity of the membranes was confirmed with simulated body fluid (SBF) treatment. From this study, the ceramic particles TiO_2_ and Na_2_Ti_6_O_13_, combined with a PCL matrix with micro/nanoparticles, enhanced cell proliferation, adhesion and antibacterial properties. The electrospun composite with Na_2_Ti_6_O_13_ can be considered viable for tissue regenerative processes.

## 1. Introduction

Electrospun membranes have been characterized for their random nanofibrous structure with interconnecting pores and a large surface-to-volume ratio. Resembling a natural extracellular matrix (ECM), these meshes have the advantage that they can provide to the cells mechanical support, and regulate cellular activities [1,2,3]. If the fibers are densely packed, they can limit the infiltration of the cells, which affects tissue recovery on wound healing in vivo [4,5], so it is mandatory to validate the configuration of the fibers for a specific application. For example, electrospun membranes have been used for bone tissue engineering applications such as membranes for maxillofacial defects [6] or guided bone regeneration (GBR). These membranes can prevent the infiltration of other tissues or bacteria, and promote bone generation with osteoprogenitor cells [7]. Some of the new advances in GBR have included adding antibacterial materials and antibiotics to avoid contamination [8,9], or adding proteins for the rapid increase of bone [10].

Many synthetic and natural polymers have been used for the fabrication of tissue-engineered membranes by an electrospinning method, such as poly(e-caprolactone) (PCL) [11,12], poly(lactide) (PLA) [13], poly(L-lactide) (PLLA) [14,15], chitosan [16] and gelatin [17,18]. Thus, for this study, PCL was selected as the biopolymer base of the membranes, because it has distinct advantages: mechanically strong, highly elastic, biodegradable, nontoxic, a good mimic of the ECM and biocompatible [19]. Chen et al. found that the diameter and uniformity of the fibers are significantly important for cellular adhesion kinetics, and these parameters were significantly higher in fibers in scaffolds on the microscale (1647 nm) compared with fibers on the nanoscale (428, 900, 1051 nm) using NIH/3T3 [11]. Also, the porosity and hydrophobicity of the membranes are crucial for cellular adhesion and proliferation [15].

The microbial proliferation on biomaterials is one of the challenges that limit the success of synthetic polymers. For this purpose, it is important to ensure its resistance to microbial attack while designing a biomaterial for clinical application [20]. By adding micro- and nanocomposites/nanoparticles [21], some scientists have found that the electrospun meshes show improved antibacterial properties and cellular proliferation [22]. Such nanoparticle properties are strongly dependent on the size, concentration, dispersion and structure. As results of several studies, nanocomposite scaffolds presented several advantages such as controllable degradation rate, enhanced bioactivity and mechanical properties, and improved scaffold structural integrity [23]. One of the nanoparticles in electrospun membranes studied is silver (Ag), whose properties make it a strong and useful antimicrobial material [8,22,24,25]. On the other hand, ceramic nanoparticles such as TiO_2_ have garnered attention because of their high chemical stability, availability and nontoxicity. Anatase, rutile and brookite are the polymorphic crystal systems of TiO_2_. Anatase has proved the most efficient species in photocatalysis, because of its higher surface area and redox ability [26]. In tissue engineering, inorganic materials such as TiO_2_ have shown excellent bacterial resistance and thermal stability over organic antibacterial materials [21,27,28,29,30,31,32,33]. Other systems studied with PCL as biopolymer for electrospinning are ZnO and Mg for their multifunctional properties, hydrophilicity and bioactivity, especially in terms of antibacterial activity [23,34].

Previous studies of PCL/Ag meshes on cell viability and cytotoxicity showed an antibacterial effect at 0.2 mM without any cytotoxic effects on hMCs cells [22]. Gupta et al. found that the PCL/TiO_2_ combination can be considered as a bioactive material that can promote apatite formation, and anatasa nanoparticles enhanced mechanical properties and cell growth, and lowered weight loss in synthetic fluids [35]. Also, this combination of a biopolymer and ceramic nanoparticle increased the NIH/3T3 cell growth and proliferation as a function of the UV exposure time [34]. Gohsal et al. found that mechanical properties were enhanced in this composite, but decreased in cell adhesion related to the concentration of nanoparticles [36]. Other researchers used TiO_2_ nanoparticles embedded in silk fibroin (SF), found that these meshes would not affect blood compatibility, and also endowed SF with bactericidal ability against *E. coli* and the ability of photocatalytic degradation of methylene blue under UV irradiation [21]. Also, studies of titania displayed its use in bone grafting, promoting the nucleation of natural hydroxyapatite (the mineral phase of bone); this process occurs when calcium and phosphorus precipitate [37,38,39]. Sodium hexatitanate (Na_2_Ti_6_O_13_) was found in superficial alkaline treatments on titania substrates to improve apatite nucleation [40].

In the present work, the antibacterial nanoparticles Ag and TiO_2_, and microparticle Na_2_Ti_6_O_13_, were added to polycaprolactone membranes produced by electrospinning and compared in terms of morphology, cellular adhesion and antibacterial properties. The obtained membranes were characterized by means of several techniques such as scanning electron microscopy (SEM), energy-dispersive X-ray spectroscopy (EDX), differential scanning calorimetry (DSC) and X-ray diffraction (XRD). Fourier-transform infrared spectroscopy (FTIR) was used to identify the nature of the interface between the polymer and the inorganic phase. Enhanced mechanical properties were found in the membranes with micro/nanoparticles. Effect on cell viability was carried out using NIH/3T3 fibroblasts. Antibacterial activity on the membranes was confirmed by using *Staphylococcus aureus*. Bioactivity of the membranes was confirmed with simulated body fluid (SBF) treatment.

## 2. Materials and Methods

### 2.1. Materials

Polycaprolactone (MW 80,000, Aldrich, St. Louis, MO, USA) (PCL), formic acid (88%, PQM, NL., Mex), acetic acid (99.7%, Aldrich, St. Louis, MO, USA), acetone (99%, CTR Scientific, N.L., Méx.), chloroform (98%, Sigma-Aldrich, St. Louis, MO, USA that was used as a solvent for PCL), and nanoparticles of TiO_2_ (≥99.5%) and silver (Ag) (99.5%, Sigma-Aldrich, St. Louis, MO, USA) were used as antibacterial particles. Na_2_Ti_6_O_13_ particles were synthesized by a sol–gel method as is described in previous work [41].

For bioactivity assays: sodium chloride (Aldrich, Saint Louis, MO, USA), sodium hydrogen carbonate (Aldrich, Saint Louis, MO, USA), potassium chloride (VETEC, Saint Louis, MO, USA), dipotassium hydrogen phosphate (Aldrich, Saint Louis, MO), magnesium chloride hexahydrate (Aldrich, Saint Louis, MO, USA), calcium chloride dihydrate (Aldrich, Saint Louis, MO, USA), sodium sulfate (Aldrich, Saint Louis, MO, USA), hydrochloric acid and tris(hydroxymethyl) aminomethane (Aldrich, Saint Louis, MO, USA) were used.

The cells NIH/3T3 ATCC ^®^ CRL-1658™ were cultured in normal medium (DMEM F-12), 5% FBS, 1% antibiotics (DIFCO Gibco^®^ Life Technologies and Sigma-Aldrich, St. Louis, MO, USA). The gram-positive bacterial strain was *Staphylococcus aureus* (ATCC 25923).

### 2.2. Polymer Solution Preparation

**Figure 1:**
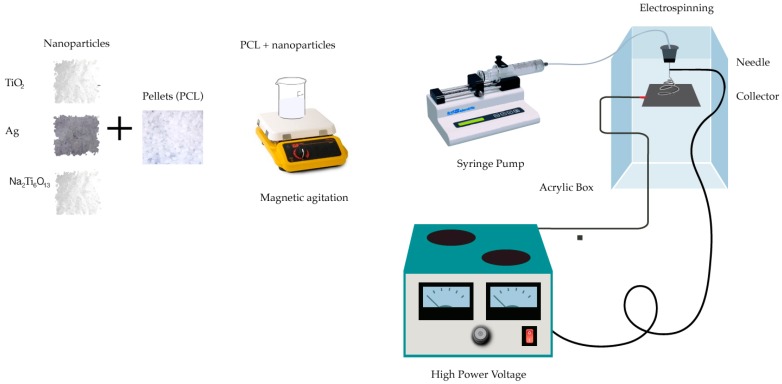
Electrospinning configuration.

PCL membranes were prepared by the electrospinning technique based on preliminary experiments (see Supplementary, Table S2). Different kinds of solvents, PCL contents, particle ratios, aging times, voltages, flow rates, the distance between needles, and collectors were selected and are registered in the Supplementary section. From those studies, a set of parameters resulted as the most accurate and repeatable in terms of fiber diameter distribution and stable jetting without dripping from the syringe tip; the beads were produced because of low polymer concentration according to previous guidelines for electrospinning processing [42]. Parameters such as concentration of PCL in chloroform of 10% (*w*/*w*), concentration of micro/nanoparticles of 1% (*w*/*w*), distance from the needle to the collector defined at 10 cm, and a flow rate of 0.3 mL/h at 20 kV were selected. All solutions were used immediately after preparation to avoid the aging effect reported in the Supplementary section. To obtain a complete dispersion, the solutions were magnetically stirred at room temperature (20° C) for 1 h. Micro/nanoparticles (Ag, TiO_2_ Na_2_Ti_6_O_13_) in a concentration of 1% (*w*/*w*) were added to the PCL solutions and delivered at a flow rate of 0.3 mL/h using a syringe pump KDS 100 (Model KDS100, KD Scientific Inc., USA), fitted with 10 mL syringes having an internal needle diameter of 0.9 mm (20 G, BD Sciences Ltd., Mexico). Solutions were electrospun at 20 kV using a high-DC-voltage power supply (Model ES20P-5W, 0–20 kV, USA) where the grounded collector was placed 10 cm away from the end of the needle. The collector was covered with commercial aluminum foil sheet approximately 20 µm thickness for collecting the solution for 10 min (Figure 1). This process was conducted at room temperature and in 50–60% humidity. During the traveling distance, the solvent gradually evaporates, and nanofibers are accumulated on the collector [15].

### 2.3. X-Ray Diffraction

Diffractograms were used to find out the crystallinity and the structure of the manufactured materials. XRD was registered in the 2.5*θ* range of 5–80° using a Siemens D-5000 (Siemens AG, Erlangen, Germany) with CuKα radiation, of which the energy was 8.04 kV and the wavelength was *λ* = 1.5418 Å with a step size of 0.05°. The applied voltage was 35 kV and the current was 25 mA.

### 2.4. Differential Scanning Calorimetry

Calorimetric measurements of the PCL doped with micro/nanoparticles were performed by means of differential scanning calorimetry (DSC; Q200, TA Instruments, New Castle, DE, USA) to prove any change of the melting temperature of the composite material in comparison with the PCL without micro/nanoparticles. The thermal behaviors of nanofibers were characterized in a temperature range from −40 °C to 250 °C at a heating rate of 10 °C/min, according to the standard test method for melting and crystallization temperatures for thermal analyses (ASTM E794), and 250 °C to −40 °C at a cooling rate of 10 °C/min with an isotherm of 1 min.

### 2.5. Fourier-Transform Infrared Spectroscopy (FT–IR)

Functional group resolution was conducted with an infrared spectrometer coupled with a Fourier-transform Perkin Elmer model Spectrum 400 (Perkin-Elmer, Shelton, CT, USA) recorded in the wavenumber range of 4000–400 cm^−1^ at room conditions. An FTIR spectroscopic analysis considered 4 samples of electrospun membranes: PCL, and the 1% *w*/*w* samples of Ag, TiO_2_ and Na_2_Ti_6_O_13_ in PCL membranes.

### 2.6. SEM and EDX

The surface morphology of the electrospun membranes was observed using a scanning electron microscopy analysis in a scanning electron microscope EVOMA25 (Carl Zeiss, Oberkochen, Germany) instrument with acceleration voltage of 5–10 kV. Samples were carefully sectioned with an approximate size of 8 mm length by 0.5 mm, and then dried in a desiccator and sputtered with carbon for 60 s in an evaporator, Quorum Q150R E. Energy dispersive X-ray (EDX) attachment of the EVOMA25 SEM analysis was carried out to confirm the presence of the particles. Fiber diameter was performed using an image visualization software ImageJ-Fiji J with the plugin DiameterJ developed by Upper Austria University of Applied Sciences.

### 2.7. Tensile Test

For the analysis of the mechanical strength of the PCL, PCL/Ag, PCL/TiO_2_ and PCL/Na_2_Ti_6_O_13_ electrospun membranes, according to Bottino et al., four samples of each kind were cut in rectangles, 5 mm gauge × 30 mm length with 0.17–0.35 mm width, and longways mounted between two grip units of the tester, leaving 3-cm gauge length for mechanical loading [41]. Cross-head speed of 10 mm/min was used for all of the specimens tested. Mechanical characterization was achieved in a universal test machine Instron 3365 with a load cell of 5 kN.

### 2.8. Effect of Fiber in Cell Viability

Cell preparation and attachment efficiency were studied on mouse fibroblasts NIH/3T3 ATCC ^®^ CRL-1658™ to determine if the Ag and ceramic nanoparticles of TiO_2_ and Na_2_Ti_6_O_13_ added on the PCL nanofibers could enhance the proliferation and viability compared to control sample (PCL). Cell adhesion and proliferation were determined by using the colorimetric MTS assay (Cell Titer, PROMEGA, Madison, Wis., USA). The mechanism behind the assay is that metabolically active cells react with tetrazolium salt in the MTS reagent to produce a soluble formazan dye which can be observed at 490 nm [43]. For this study, cells were cultured in a 12-well plate (Corning, N.Y., USA) at 37 °C + 5% CO2 with PCL, PCL/Ag, PCL/TiO_2_ and PCL/Na_2_Ti_6_O_13_ which were sterilized by UV irradiation for 20–30 min. NIH/3T3 cells were seeded at a concentration of 1 × 10^5^ cells/mL in DMEM F-12, SBF 5% and 1% of antibiotics (DIFCO Gibco^®^ Life Technologies and SIGMA-Aldrich, St. Louis, MO, USA) for 72 h at 37 °C, 5% CO_2_, and controlled humidity. After this, Cell TITER was added in the order that the supplier instructed, and incubated for 1 h at 37 °C, 5% CO_2_, and controlled humidity. The samples were measured in a spectrophotometric plate reader, ELx808 (BioTek, Winooski, VT, USA), at 490 nm. One-way anova and post-hoc Tukey’s test method were used to determine the statistical significance. Differences were considered significant at *α* = 0.05.

### 2.9. Cell Adhesion

For evaluating the cell adhesion of the NIH/3T3 fibroblasts, in the fibers of PCL, PCL/Ag, PCL/TiO_2_ and PCL/Na_2_Ti_6_O_13_ we used a blue stain, DAPI (4′,6-diamidino-2-phenylindole). The study followed the same process of cell growth and proliferation as the cell viability assay. The cells attached to the membranes were separated and processed in a new well, and analyzed according to the DAPI protocol reported by Brad Chazotte [44]. Finally, the fibers were observed in an optical microscope, Carl Zeiss AXIOVERT 200 (Carl Zeiss, Oberkochen, Germany).

### 2.10. Antibacterial Assay

The antibacterial activity of the electrospun membranes against the gram-positive bacteria *Staphylococcus aureus* (ATCC 25923) was studied under UV exposure using the drop-test method [21,45]. From stock, *Staphylococcus aureus* colonies were colocated in Mueller–Hinton agar plate (cultured at 37 °C for 18 h) and suspended in sterilized PBS solution to adjust the turbidity to the McFarland nephelometer to reach approximately the concentration of bacteria corresponding to 1.5 × 10^8^ bacteria/mL. The drop-test method was performed using only one sample, after the exposition of 20 min with a UV lamp. A volume of 100 µL of PBS solution containing *Staphylococcus aureus* bacteria was added dropwise to the surface of each 1.5 × 1.5 cm fiber (PCL, PCL/Ag, PCL/TiO_2_ and PCL/Na_2_Ti_6_O_13_). The samples were irradiated under UV-A (*λ* = 254 nm) light for 20 min at room temperature (20 °C), and then the samples were seeded in Mueller-Hinton agar plates for 24 h at 37 °C in a glass incubator to evaluate the bacterial growth. The *Staphylococcus aureus* was evaluated by the plating technique.

### 2.11. Bioactivity Test

Bioactivity test was performed with PCL/TiO_2_ and PCL/Na_2_Ti_6_O_13_ membranes, where the microparticles were immersed to prove the generation of hydroxyapatite through time. This study was evaluated by soaking for 72 h in simulated body fluids at 37 °C. Simulated body fluids were prepared to dissolve different salts from the Kokubo recipe for SBF [46], and pH was adjusted to 7. Samples were dried and covered with carbon for SEM studies.

## 3. Results and Discussion

### 3.1. X-Ray Diffraction

**Figure 2:**
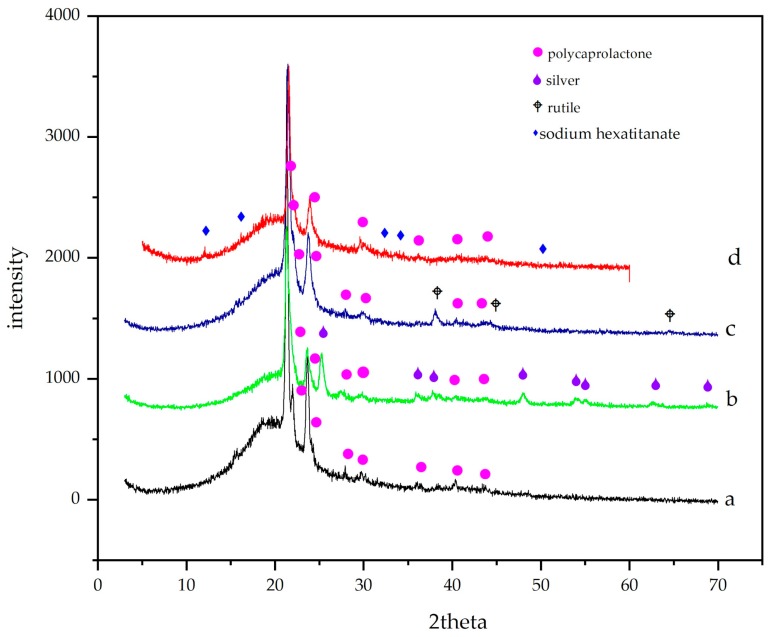
XRD studies of the electrospun membranes of (**a**) PCL, (**b**) PCL/Ag, (**c**) PCL/TiO_2_, and (**d**) PCL/Na_2_Ti_6_O_13_, histograms at the same magnification.

Characteristic PCL intensities (21.31°, 23.71°) were observed in XRD data (Figure 2a), together with a large bump between 15° and 25° due to semicrystalline PCL. Because of the amount of particles (1%), only peaks with the highest intensities were observed in each sample. In Figure 2b, typical highest reflections of titanium dioxide are shown at 21.31°, 23.71° and 48.03° (JCPDS 00-004-0783) [35]. In Figure 2c, two phases were detected: PCL and Ag at 38.13°, 44.43° and 64.54° (JCPDS 04-0783). In Figure 2d, besides PCL signals, Na_2_Ti_6_O_13_ showed higher peaks at lower grades (12.16°, 16.20° and 32.42° JCPDS 01-073-1398). The polycaprolactone bump (21.45° to 21.70°) is displaced slightly to the right due to the interactions with titania crystals. Also observed were the anatase and rutile signals, which are broadened probably due to interactions between hydrophilic TiO_2_ and polar PCL, as is mentioned by Gupta et al. [47].

### 3.2. Differential Scanning Calorimetry

Thermal analyses by DSC were performed in order to investigate thermal properties of the electrospun membranes doped with micro/nanoparticles. Results of DSC curves of the PCL, PCL/Ag, PCL/TiO_2_ and PCL/Na_2_Ti_6_O_13_ samples are summarized in Table 1. By testing the membranes with an exothermic and endothermic range of temperatures, the membranes performed similarly because there was a low concentration of the micro/nanoparticles. The electrospun membranes of PCL/Ag exhibited the lowest endothermic peak at 55.57 °C, which indicates the melting point (Tm), in comparison with the electrospun membranes of PCL/TiO_2_ (60.85 °C), and PCL/Na_2_Ti_6_O_13_ (57.81 °C). Ag is a metal with strong thermal conductivity as a result of free electrons located in its lattice, in contrast to polymers and ceramic materials such as the PCL, TiO_2_ and Na_2_Ti_6_O_13_, which lack free electrons, making them nonconducting materials. Thus, metal particles slightly reduce the melting point. By comparing the two ceramics, the PCL/Na_2_Ti_6_O_13_ membranes exhibited a higher thermal conduction because they have a higher gap distance in their crystal structure [41].

### 3.3. Fourier-Transform Infrared Spectroscopy (FT–IR)

**Figure 3:**
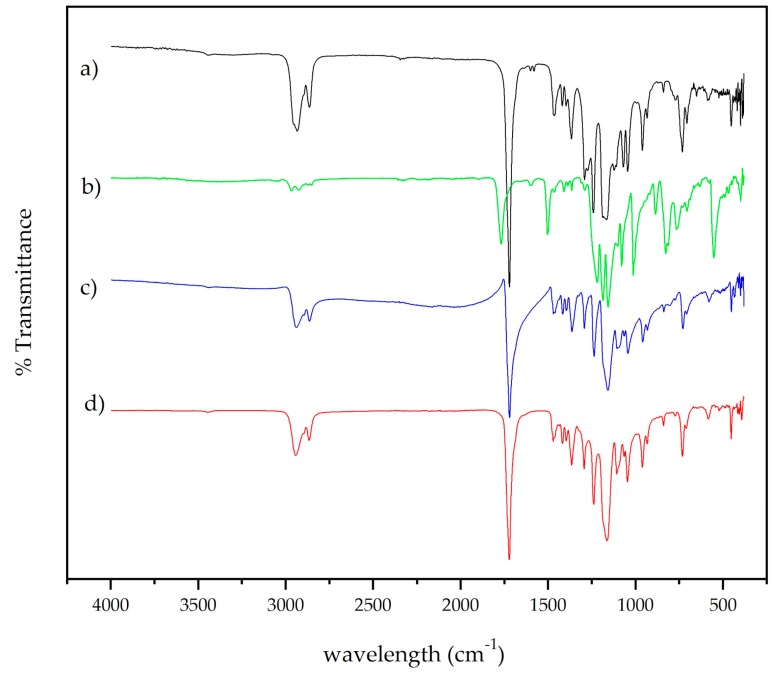
FTIR spectra of electrospun membranes of (**a**) PCL, (**b**) PCL/Ag, (**c**) PCL/TiO_2_, and (**d**) PCL/Na_2_Ti_6_O_13_.

FTIR analysis is shown in Figure 3, with spectra of PCL membrane, PCL with titanium dioxide powder, PCL membrane with silver, and PCL membrane with sodium hexatitanate powder. In the near IR, typical bands were found in the spectra (Figure 3a). At 2936 and 2861 cm^−1^, methylene groups were found, and at 1724 cm^−1^ there was a band slightly displaced due to the presence of carbonyl. The carboxylic acid bend band (C–O–H) in-plane was at 1462 cm^−1^ and at 933 cm^−1^ out-of-plane. C–O strong stretching bands were observed at 1300–1000 cm^−1^. A typical band located at 732 cm^−1^ corresponded to the scissor-like bending mode of methylene groups. In Figure 3b, the carbonyl band at 1764 cm^−1^ was displaced due to the interaction with Ag particles; also at 540 cm^−1^, a broad and strong band of Ag–O was observed. Metal–oxygen bands are typically seen at lower wavenumbers; in this case, for titanium–oxygen (Figure 3c,d), these small bands were placed at 452 and 404 cm^−1^ in both cases with content of Ti–O functional groups.

### 3.4. SEM and EDX

**Figure 4:**
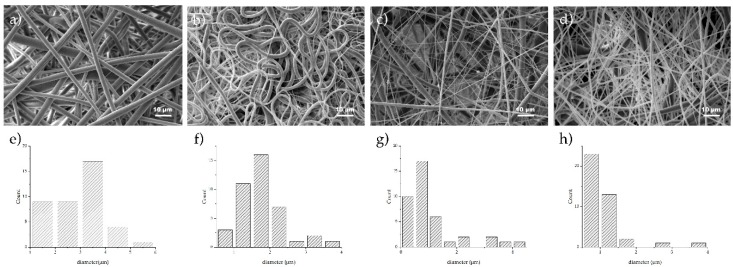
SEM photomicrographs of electrospun samples and diameter distribution; (**a**,**e**) PCL; (**b**,**f**) PCL/1% Ag; (**c**,**g**) PCL/TiO_2_; and (**d**,**h**) PCL/Na_2_Ti_6_O_13_. Fiber distribution is observed in each graph, *n* = 40.

Photomicrographs of electrospun samples were taken (Figure 4), and well-defined and aligned fibers of PCL in a random distribution were observed; average diameter fiber was 2.99 μm with a standard deviation of 1.02 μm. Fibers showed good metallic particle distribution with a silver particle size around 100 nm (Figure 4b). Surface fibers were smooth with homogeneous diameter (standard deviation was 1.76 μm and diameter average was 0.64 μm). When ceramic particles were added, meshes showed differences in diameter due to the particle size. In Figure 4c, average titania particle diameter was 21 nm, and in fibers, clusters trapped in the PCL matrix were observed. This fiber had a diameter of 1.08 μm with a standard deviation of 0.97 μm with *n* = 40 measurements. Comparing Figure 4a with Figure 4b,c, average diameters suggested that deposition is governed by the higher attraction between the particles and the grounded collector stretching the fiber during the process. PCL/Na_2_Ti_6_O_13_ fibers were randomly collected, and were thicker, and due to the preparation method, ceramic particles were nonhomogeneous and with a larger size (Figure 4d). The hexatitanate sodium membranes presented an average diameter of 1.01 μm and standard deviation of 0.54 μm. Consistent results in diameter distribution of the fibers were found in each of the PCL systems where diameter averages varied from 0.64–2.99 μm, and uniformity was presented ideal for cell adhesion [11]. Distribution of the diameter size was analyzed using ImageJ, and it is presented in Figure 4e–f.

### 3.5. Tensile Test

Table 1 shows mechanical strength which consists of composition, fiber diameter and mesh density. All membranes enriched with particles displayed a superior mechanical strength than PCL membranes (Table 1, Supplementary Figure S4). PCL/Na_2_Ti_6_O_13_ membranes raised strength from 0.648 (PCL) to 1.55 MPa. In turn, nanoparticles considerably enhanced strength to 2.50 MPa in PCL/Ag and to 6.16 MPa in PCL/TiO_2_ membranes. The pristine PCL sample was reinforced, increasing from 1.5 MPa to 6.19 MPa, with titania (Table 1); the reinforcement process is comparable to those used with other meshes [35,48]. The highest deformation was observed for the PCL/Ag matrix with 900%, in comparison with 160% deformation shown by PCL. The deformation gradient between PCL/Ag and TiO_2_ in contrast with PCL/Na_2_Ti_6_O_13_ could be related to a better homogenization of nanoparticle size versus microparticle size obtained by the sol–gel process (3–7 μm) (see Supplementary Section Figure S1). Ceramic matrixes displayed classical fragile behavior, however, in contrast, PCL/Ag showed good plastic properties. Although the sodium hexatitanate matrix had a lower strength in comparison with PCL/TiO_2_, it had a good strain (400%).

### 3.6. Cell Viability and Adhesion

**Figure 5:**
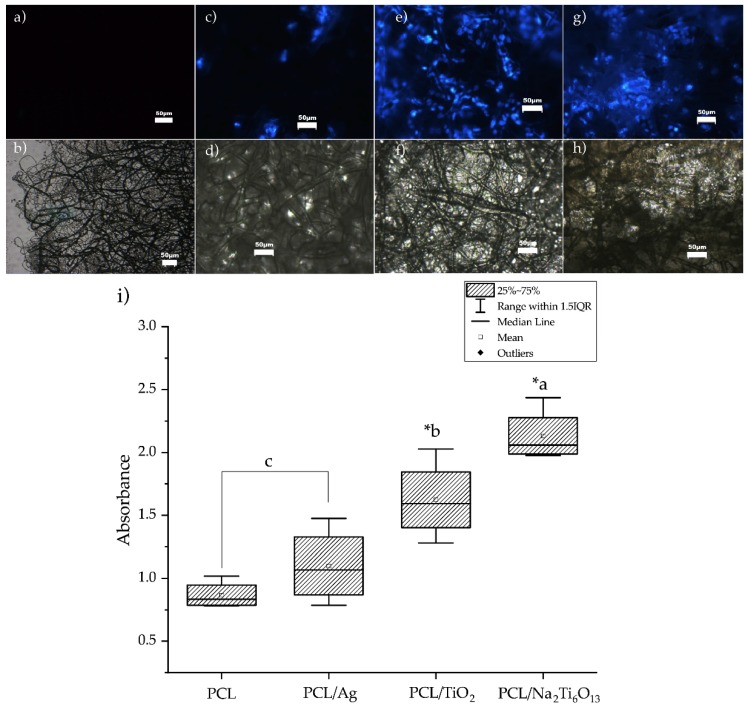
Microscopic observation for NIH-3T3 cells stained with DAPI, objective 20×. In a fluorescence microscope can be observed the cells collocated and attached on the electrospun membranes of (**a**) PCL mesh, (**c**) PCL/Ag, (**e**) PCL/TiO_2_ and (**g**) PCL/Na_2_Ti_6_O_1__3_. (**b**,**d**,**f**,**h**) show cells attached on the electrospun membranes in brightfield illumination. (**i**) Result of the MTS assay. Analysis with one-way anova and post-hoc Tukey comparisons showed a significant difference between the membranes with micro/nanoparticles. *a = α < 0.000 vs c, *b = (α < 0.05) vs c.

The DAPI staining test confirmed that PCL and PCL/Ag fibers did not promote cell adhesion (Figure 5a,c). Nanofibers of PCL/TiO_2_ and PCL/Na_2_Ti_6_O_13_ had a high number of cells attached (Figure 5e,g). Cell viability assays demonstrated that the PCL/Na_2_Ti_6_O_13_ membrane allowed the best proliferation of NIH/3T3 cells, followed by PCL/TiO_2_ (α < 0.05). PCL and PCL/Ag showed poor cell viability (Figure 5i).

### 3.7. Antibacterial Assay

A preliminary antibacterial assay performed in a plate that received no UV treatment had countless *S. aureus* colonies. UV treatment of the ceramic micro/nanoparticles caused bacterial death, since the colonies dispensed in the membranes of PCL/TiO_2_ and PCL/Na_2_Ti_6_O_13_ showed no growth (100% inhibition, see Figure S6 in Supplementary section). The plate for PCL/Ag^+^ bacteria had only around 10% inhibition due to the low Ag concentration used in this study. Additional tests are required to obtain a complete curve at various times of UV light treatment [19].

### 3.8. Bioactivity Test

**Figure 6:**
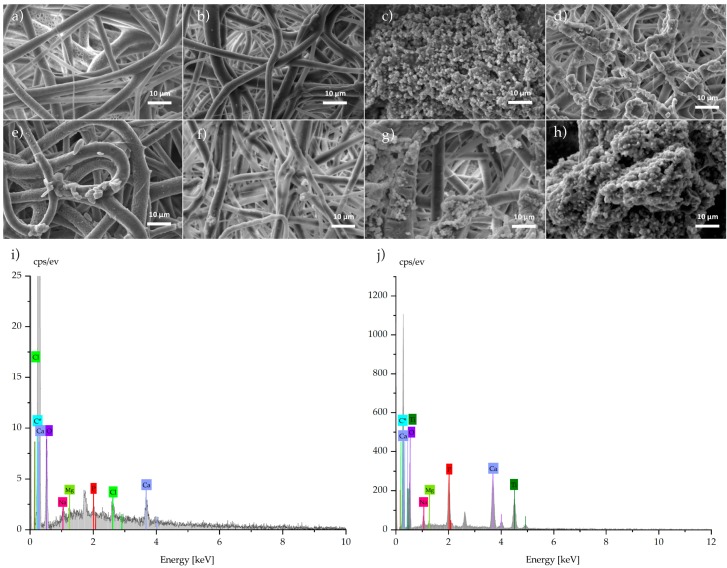
Micrographs of meshes soaked for 14 days: (**a**) PCL, (**b**) PCL/Ag, (**c**) PCL/TiO_2_, and (**d**) PCL/Na_2_Ti_6_O_13_; meshes soaked for 21 days: (**e**) PCL, (**f**) PCL/Ag, (**g**) PCL/TiO_2_, and (**h**) PCL/Na_2_Ti_6_O_13_;and EDS spectra of samples soaked for 21 days: (**i**) PCL/TiO_2_, and (**j**) PCL/Na_2_Ti_6_O_13_.

Previous studies have indicated that TiO_2_ [30,49] and Na_2_Ti_6_O_13_ [50,51] are bioactive ceramics promoting hydroxyapatite formation. In Figure 6, samples soaked for 14 days did not show a large change in the substrate (Figure 6a,b), while in contrast, those samples with titanium content showed clusters with a concentration of phosphorus and calcium, and were rounded apatite-like clusters (Figure 6c,d). The morphology of the cluster depends on various parameters like content of Ca and P in solution, pH, temperature and so on, as Jiang et al. reported for round aggregates [52]. The images in Figure 6e,f suggest that early stages of apatite formation occur in the surface of mesh which is more exposed to the interchange of solvated ions of SBF. The diffusion could be slower in the bulk, while on the surface, an easy ion interchange may promote the nucleation of calcium and phosphorous particles. EDS spectra were collected for samples (Figure 6i,j), and a high concentration of calcium and phosphorous was detected in spherical formations.

Images show that for samples at 21 days soaked in fluids, the formation of clusters with a high content of calcium and phosphorous occurred with ratio of 1.70 (Figure 6i) and 1.46 (Figure 6j), close to ratio expected for natural apatites (1.64). The infrared spectrum confirms the presence of classical bands of phosphorus in the apatite compound only in samples with titanium content.

**Figure 7:**
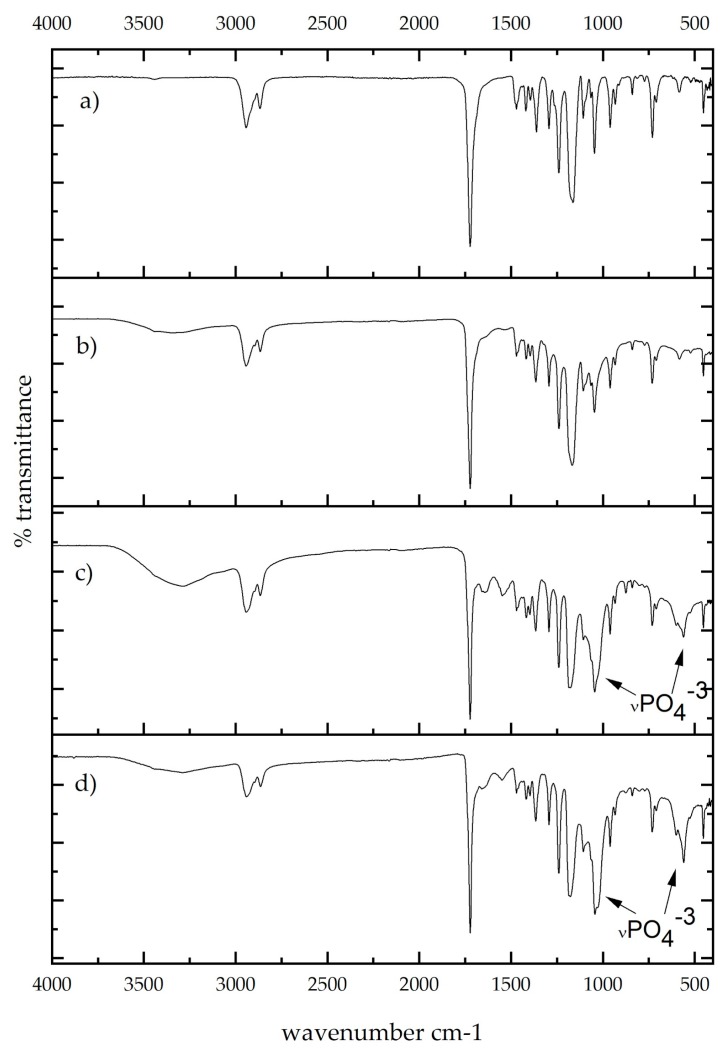
Infrared spectra for samples soaked for 21 days: (**a**) PCL, (**b**) PCL/Ag, (**c**) PCL/TiO_2_, and (**d**) PCL/Na_2_Ti_6_O_13_.

Soaked samples were analyzed with Fourier-transform infrared spectroscopy. In Figure 7, spectra show interactions of polycaprolactone and silver meshes after 21 days soaked in simulated body fluids. The spectra of PCL and PCL with 1% silver present similar bands to those without the immersion in fluids. For Figure 7c,d), different bands have developed; at 557 cm^−1^ and a broad band at 1046 cm^−1^ are bands related to bending and stretching modes of the P–O bond [53]. In this case, this study confirms the bioactivity previously found in this kind of material.

## 4. Conclusions

In summary, PCL membranes with micro/nanoparticles were successfully electrospun with fibers with average diameters around 1.18 and 1.8 μm for silver and TiO_2_, respectively. Fiber diameter for hexatitanate increased in comparison to PCL due to the different particle size obtained from the synthesis method. In all cases, reinforcing PCL with micro/nanoparticles enhanced tensile strength and led to retention of good deformation properties. Cell viability studies showed a good compatibility, especially for sodium hexatitanate samples in ceramic matrixes. Further research on biocompatibility with osteoblastic cells is needed. This study demonstrated that the PCL/Na_2_Ti_6_O_13_ and TiO_2_ fibers obtained by the electrospinning technique had apatite-forming ability and allowed cell proliferation.

## Figures and Tables

**Table 1:** Differential scanning calorimetric data and mechanical properties obtained by analyzing the electrospun membranes of PCL, PCL/Ag, PCL/TiO_2_ and PCL/Na_2_Ti_6_O_13_. * *n* = 4.

Samples	*t_m_* (°C)	Tensile Strength* (MPa)	Strain at Break (%)
PCL (control)	58.88	0.66 ± 0.13	152.40 ± 0.758
PCL/Ag	55.67	2.51 ± 0.13	901.65 ± 0.001
PCL/TiO_2_	60.85	6.19 ± 0.72	190.18 ± 0.001
PCL/Na_2_Ti_6_O_13_	57.87	1.55 ± 0.24	472.70 ± 0.013

## Supplementary Materials

The following are available online at http://www.mdpi.com/2077-0375/9/1/12/s1, **Figure S1.** Size particle mean in a SEM photomicrograph and respective length distribution graph. **Table S1.** Solvent properties. **Figure S2**. Viscosity behavior through time, 10%PCL in solvent *w*/*w*. All concentration content was adjusting during the study. **Table S2.** Electrospun parameters (Applied Electric Voltage and Distance between collector and needle), polymer concentrations, micro-nanoparticles concentrations. **Table S3.** Morphology and diameter fiber at different parameters. **Figure S3.** DSC of electrospun membranes of (a) PCL, (b) PCL/Ag, (c) PCL/TiO_2_, and (d) PCL/Na_2_Ti_6_O_13_. **Figure S4.** Mechanical performance of electrospun membranes of (a) PCL, (b) PCL/Ag, (c) PCL/TiO_2_ and (d) PCL/Na_2_Ti_6_O_13_. Mechanical characteristics are shown in Figure S4b, c, and d developed a better performance than a. S4b increase maximum strength and 200% of strain. A fragile behavior was evolved by PCL/TiO_2_ and the best strength (~6 MPa) while observed the maximum strain of the system. **Figure S5**. Microscopic observations for NIH-3T3 cells in PCL/Na_2_Ti_6_O_13_, stained with DAPI, objective 32×. (a) PCL/Na_2_Ti_6_O_13_ in brightfield illumination, (b) PCL/Na_2_Ti_6_O_13_ in fluorescent field and (c) Merge of both. **Figure S6**. Microbiological assay. (a) PCL fibers without treatment of UV light, (b) PCL fiber with Ag and (c) PCL fiber with TiO_2_ and (d) PCL fiber with Na_2_Ti_6_O_13_.
