# Supplementary material for: Electrospun Polycaprolactone Fibrous Membranes Containing Ag, TiO2 and Na2Ti6O13 Particles for Potential Use in Bone Regeneration
Source: Membranes (Basel). 2019 Jan 10;9(1):12. doi: 10.3390/membranes9010012 (PMC6359384; doi:10.3390/membranes9010012)
Supplement: Supplementary file 1 [file membranes-09-00012-s001.pdf]

Supplementary material

Electrospun Polycaprolactone Fibrous Membranes Containing Ag, TiO_2 and $Na_2Ti_6O_{13}$ Particles for Potential Use in Bone Regeneration

Erick Ramírez-Cedillo [1,3], Wendy Ortega-Lara [1,3,*], María R. Rocha-Pizaña [1,2], Janet A. Gutierrez-Uribe [1,2], Alex Elías-Zúñiga [1,3] and Ciro A. Rodríguez [1,3]

1 Tecnologico de Monterrey, Escuela de Ingeniería y Ciencias, Av. Eugenio Garza Sada #2501 Sur, Monterrey, NL 64849, Mexico; A00806274@itesm.mx (E.R.-C.); mrochap@tec.mx (M.R.R.-P.); jagu@tec.mx (J.A.G.-U.); aelias@tec.mx (A.E.-Z.); ciro.rodriguez@tec.mx (C.A.R.)

2 Tecnologico de Monterrey, Escuela de Ingeniería y Ciencias, Vía Atlixcáyotl 2301, Reserva Territorial Atlixcáyotl, Puebla 72453, México

3 Laboratorio Nacional de Manufatura Aditiva y Digital (MADIT), Autopista al Aeropuerto, Km., 9.5, Calle Alianza Norte #100, Parque PIIT, Apodaca, N.L. 66629, México.

* Correspondence: wlortega@tec.mx; Tel.: +52-8358-2000

Received: 1 December 2018; Accepted: 3 January 2019; Published: date

S.1. Size Diameter of $Na_2Ti_6O_{13}$

Particles obtained by sol-gel method were observed in a scanning electronic microscopy analysis (VEGA3 TESCA) with acceleration voltage of 5–10 kV. Size diameter mean is 3.3095 μm and standard deviation is 3.069 of a *n* = 20. Figure S1.

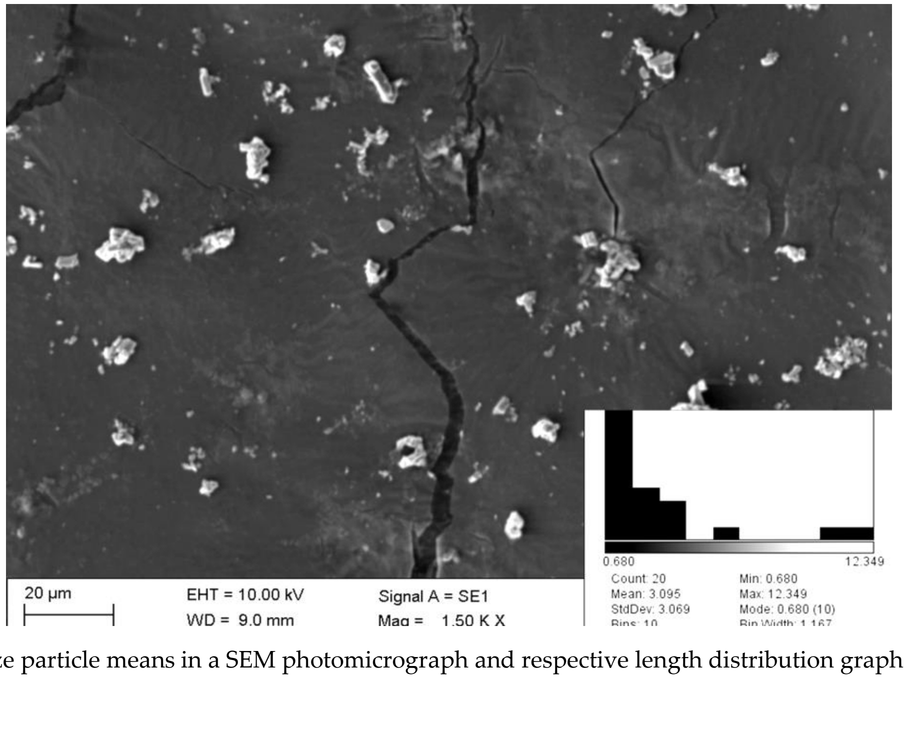

Figure S1. Size particle means in a SEM photomicrograph and respective length distribution graph

S.2. Viscosity Study

The viscosity of several systems was observed through time. Polycaprolactone (PCL) at 10% w/w was dissolved in different solvents that were reported as substances with the capability to dissolve PCL, furthermore possessed good conductivity for electrospinning technique as is observed in Table S1. Systems were elected according to those suggested by the spinnability-solubility map of Luo and cols [1]. Different solvent were studied to observe spinnability and thinner fibers diameter.

Table S1. Solvent properties.

Solvent	M_w	*Bp* (°C)	*ε* at 20 °C	Dipole	Electrical Conductivity (Sm^{-1})	Surf. Tension at 20°C mNm^{-1}	Abs Viscosity (mPa s)	Supplier, Purity
Acetic acid (AA)	60	118	6.2	1.7	6.0×10^{-7}	27.4	1.13	Aldrich, 99+
Acetone (A)	58	56	20.6	2.9	5.0×10^{-7}	23.3	0.33	CTR, 99.6%
Chloroform (C)	119	61	4.8	1.15	1×10^{-8}	27.16	0.57	Aldrich/ Vetec, 99%
Formic Acid (FA)	46	100.8	58	1.41	6.4×10^{-3}	37.67	1.78	Monterrey Chem Prod, 88.8%

S.3. Aging Studies

Viscosity studies were assessed to obtain information about viscosity through time. The results show that acetone system exhibits a more uniform viscosity during the study (Figure S1). However the evaporation rate makes the system unstable, during all process, the system requires the addition of acetone trying to maintain concentration. The systems AA-FA and Chloroform observed pronounced curves due to pellet dissolution in the solvent meanwhile acetone spends at least five hours. Chloroform shows the highest viscosity even at the end of the compilation, AA-FA shows a higher viscosity than acetone and results stable through the experiment. The recommendation to select solvents different to Chloroform is using immediately when a homogenous solution is obtained because viscosity will drop down drastically. Viscosity, one of the principal parameters for getting a proper diameter size and reproducibility.

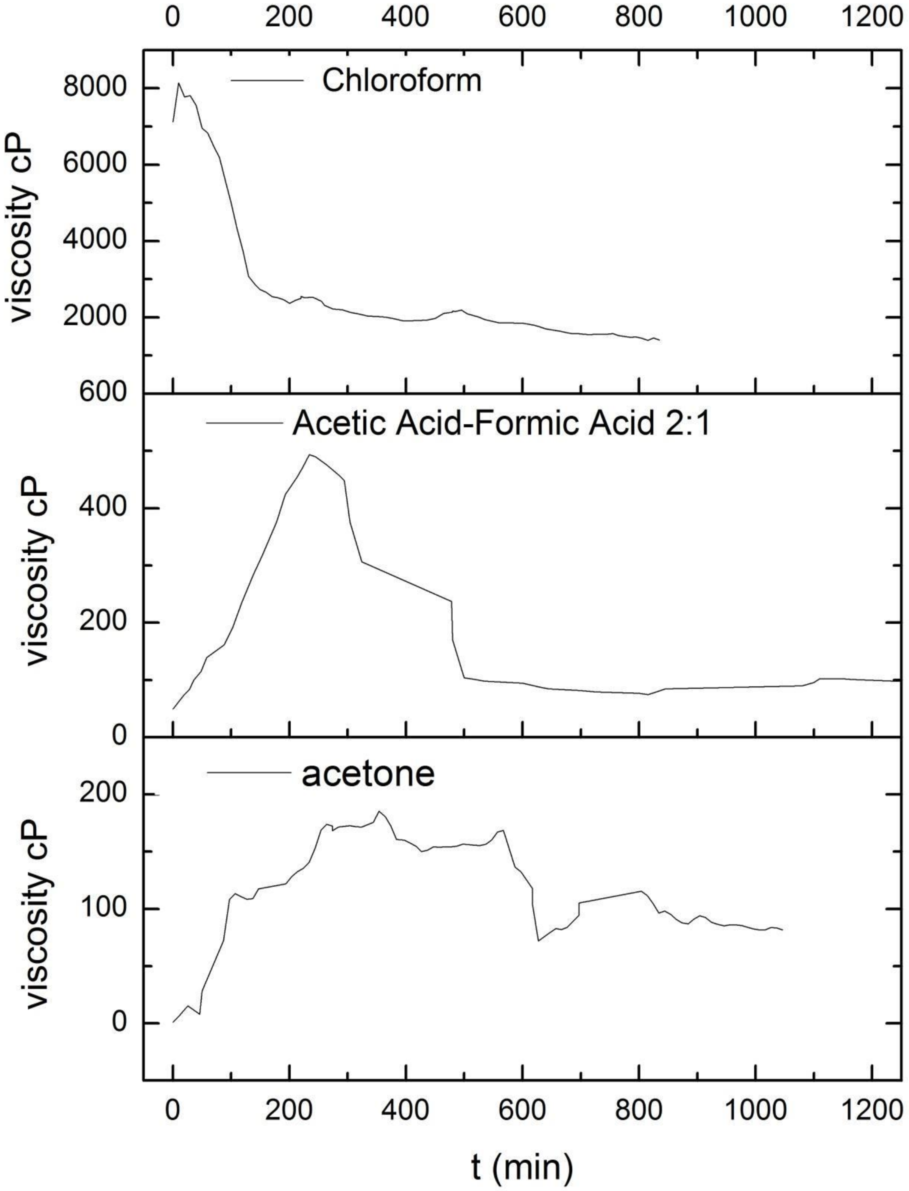

Figure S2. Viscosity behavior through time, 10%PCL in solvent w/w. All concentration content was adjusting during the study.

Table S2. Electrospun parameters (Applied Electric Voltage and Distance between collector and needle), polymer concentrations, micro-nanoparticles concentrations.

Voltage (kV)	Distance Between the Collector and the Needle (cm)	Polycaprolactone (PCL) (% w/w)	Ag, TiO_2 and $Na_2Ti_6O_{13}$ (% wt)	Flow Rate (ml/h)
12.5	8.5	5	1	1
15	13.5	10		2
17.5		15		

S.3. Microscopy Analysis of Different Solvent Systems

SEM micrographs are shown in Table S2; images indicates all systems were capable of obtaining fine fibers, without bead formation. In the table, electrospinning parameters are displayed for each sample. The percentage of PCL in a solvent was fixed at 10% w/w. Dissolution time to achieve homogeneity, reproducibility, and handling was evaluated to attain a stable system for particle addition. The thinnest diameter was obtained for FA-AA system at 10% PCL w/w (0.35 μm), and a narrow dispersion was displayed by AA-FA 1:3 (0.41–1.20 μm). Although best results could be reached with FA-AA, Chloroform system is highly stable due to its low volatility furthermore dissolving time was shorter than other solvents as could be studied in the viscosity analysis section. Acetone system inconvenience was a rapid rate of evaporation that changes viscosity drastically in a shorter lapse (approx. 15 minutes). A-FA system shows the slowest evaporation rate, samples displays fibers interconnected and losing their form merging with other fibers.

Table S3. Morphology and diameter fiber at different parameters.

System	Conc.	Distance/ Voltage	Rate	Morphology—504×	Measurements	Diameter Average
FA-AA 1:3	10% PCL	8.5 cm/ 15kV	2ml/h	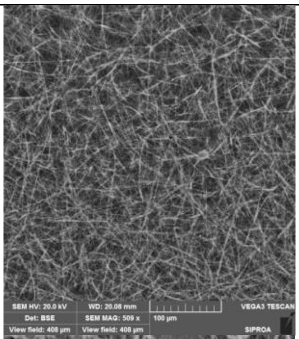	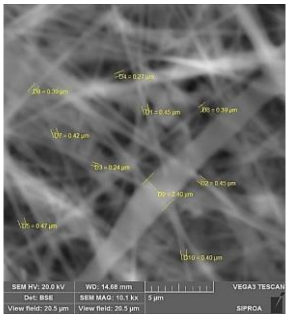	0.24–2.40
A-FA 1:3	15% PCL	8.5 cm/ 20kV	2ml/h	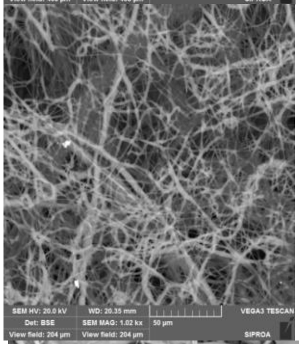	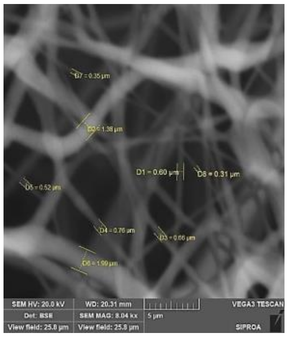	0.31–1.99
A-FA 1:3	15% PCL	8.5 cm/ 10kV	2ml/h	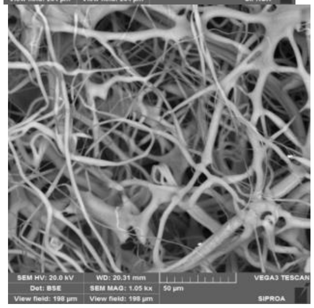	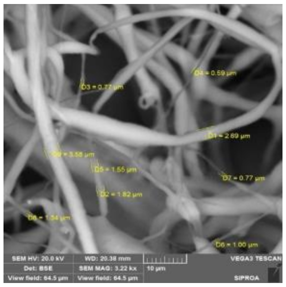	0.59–3.58

A-FA 1:3	15% PCL	8.5cm/15kV	2ml/h	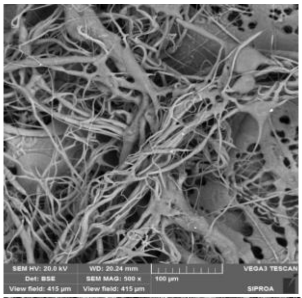	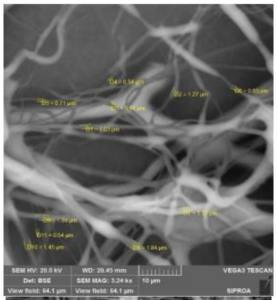	0.54–1.84
AA-FA 1:3	15% PCL	8.5 cm/ 15kV	2ml/h	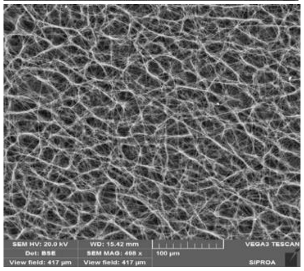	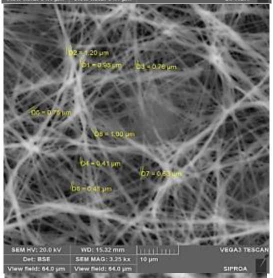	0.41–1.20
FA-AA 1:3	15% PCL	8.5 cm/ 15kV	0.5ml/h	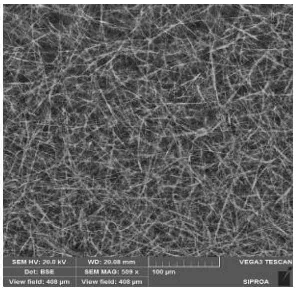	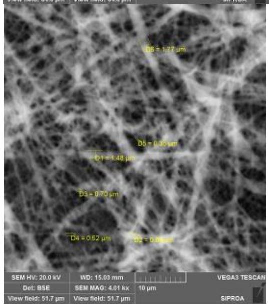	0.35–1.77
Chloroform	10% PCL	8.5 cm/ 10kV	0.5ml/h	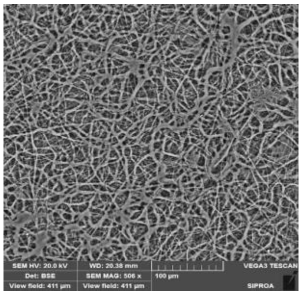	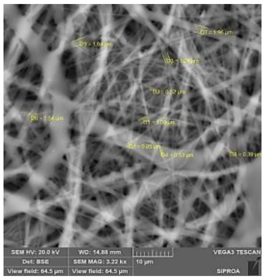	0.39–1.96
Chloroform	10% PCL	8.5 cm/ 20 kV	0.5ml/h	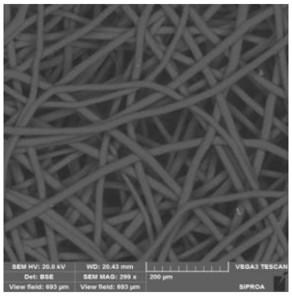	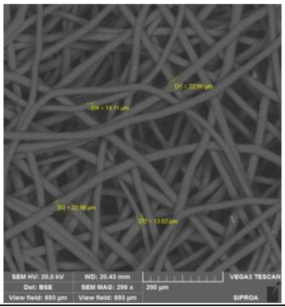	13.82–22.96

Table S3 Con.

System	Conc.	Distance	Rate	Morphology	Measures	Diameter Average
Chloroform	15% PCL /1% $Na_2Ti_6O_{13}$	8.5cm/ 20KV	5ml/ h	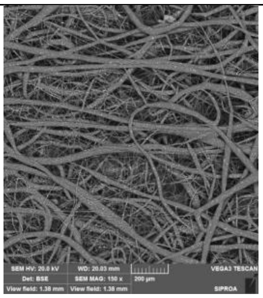	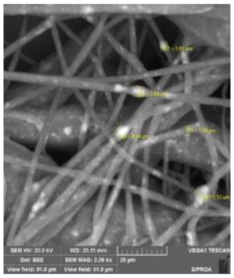	3.01–4.98
					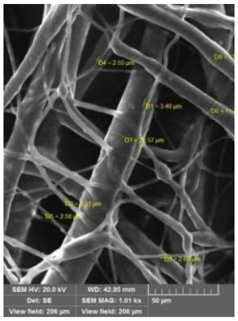	
Chloroform	10% PCL/ TiO_2	8.5cm/ 18kV	0.5 ml/h	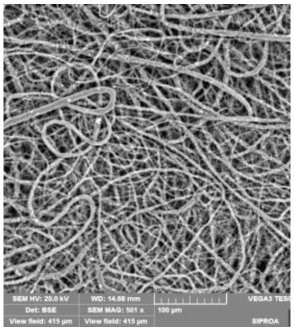	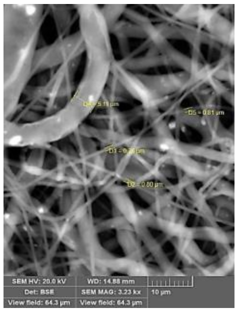	0.76–0.94
Chloroform	10% PCL /Ag	8.5 cm/ 20kV	2 ml/h	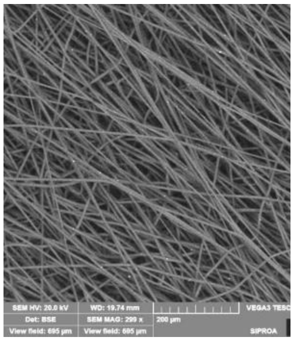	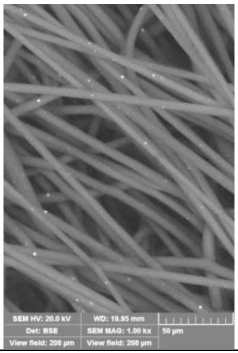	0.59–3.58

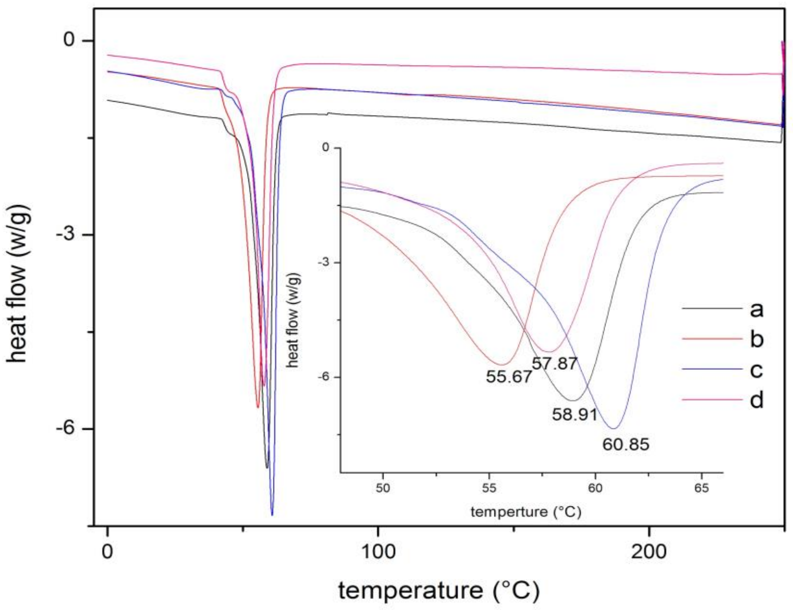

Figure S3. DSC of electrospun membranes of (**a**) PCL, (**b**) PCL/Ag, (**c**) PCL/TiO_2, and (**d**) PCL/$Na_2Ti_6O_{13}$

In this image melting point displacement was observed, particle reinforcement beat down the melting point due to metallic and semiconductor particles.

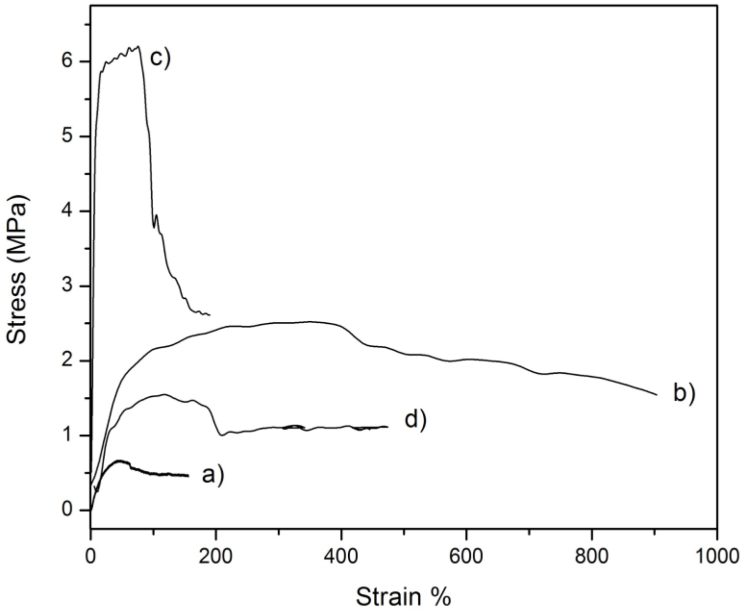

Figure S4. Mechanical performance of electrospun membranes of (**a**) PCL, (**b**) PCL/Ag, (**c**) PCL/TiO_2 and (**d**) PCL/$Na_2Ti_6O_{13}$. Mechanical characteristics are shown in figure S4b, c, and d developed a better performance than a. S4b increase maximum strength and 200% of strain. A fragile behavior was evolved by PCL/TiO_2 and the best strength (~6 MPa) while observed the maximum strain of the system.

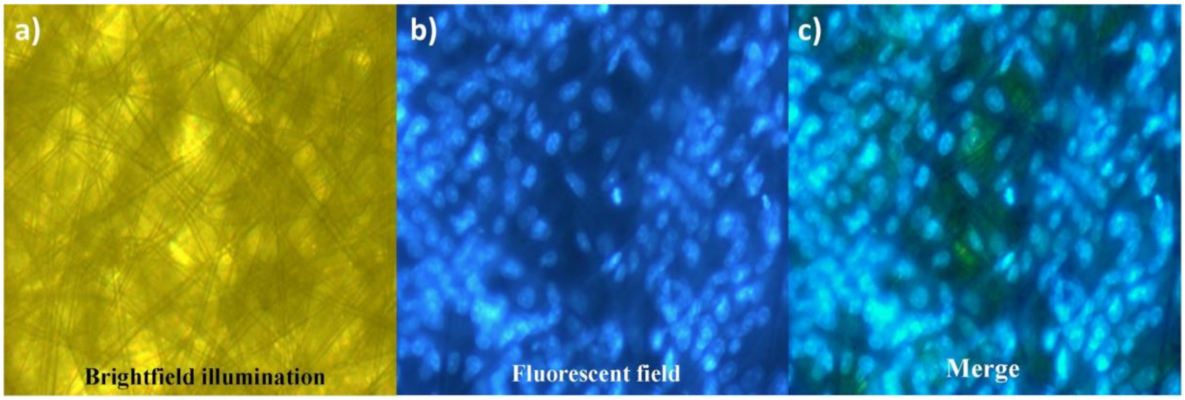

Figure S5. Microscopic observations for NIH-3T3 cells in PCL/$Na_2Ti_6O_{13}$, stained with DAPI, objective 32×. (**a**) PCL/ $Na_2Ti_6O_{13}$ in brightfield illumination, (**b**) PCL/$Na_2Ti_6O_{13}$ in a fluorescent field and (**c**) Merge of both.

(a) **(b)**

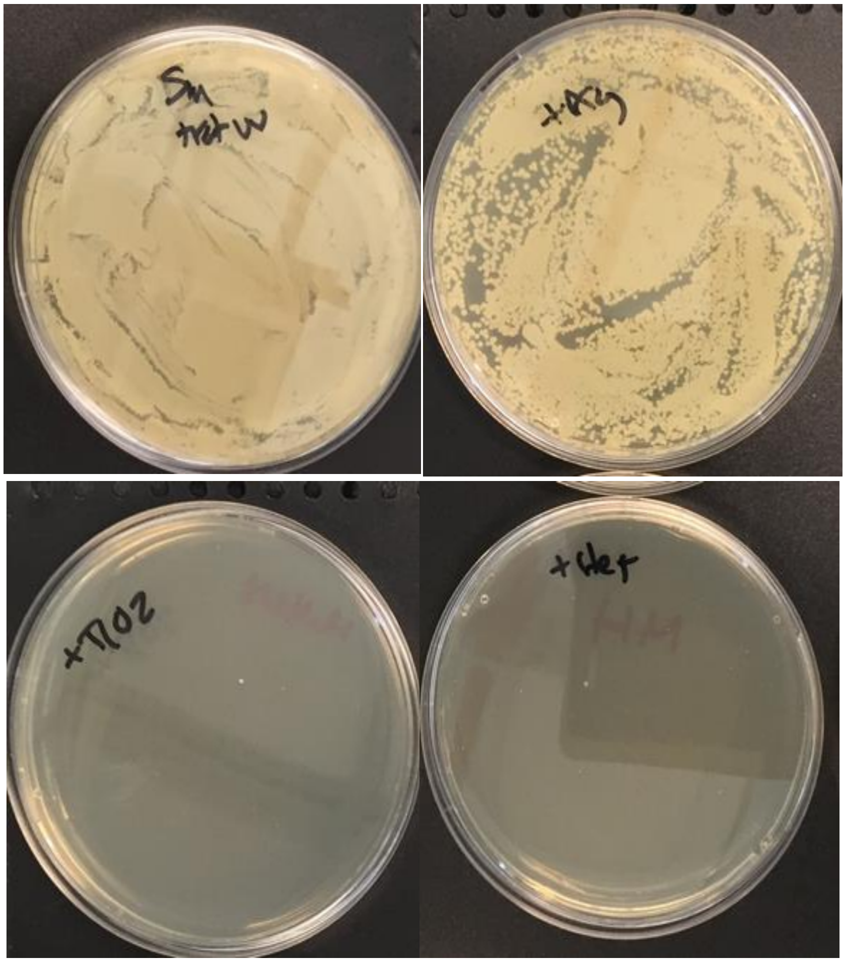

(d) **(e)**

Figure S6. Microbiological assay. (**a**) PCL fibers without treatment of UV light, (**b**) PCL fiber with Ag and (**c**) PCL fiber with TiO_2 and (**d**) PCL fiber with $Na_2Ti_6O_{13}$.

Figure S6 showed antibacterial properties in S6c and S6d, this suggests that photocatalytic characteristics played an important role in bactericide nature.

References

1. Luo, C.J.; Stride, E.; Edirisinghe, M. Mapping the Influence of Solubility and Dielectric Constant on Electrospinning Polycaprolactone Solutions. *Macromolecules* **2012**, *45*, 4669–4680.
